# Resveratrol Induces Cell Cycle Arrest and Apoptosis in Malignant NK Cells via JAK2/STAT3 Pathway Inhibition

**DOI:** 10.1371/journal.pone.0055183

**Published:** 2013-01-25

**Authors:** Ly Quoc Trung, J. Luis Espinoza, Akiyoshi Takami, Shinji Nakao

**Affiliations:** 1 Cellular Transplantation Biology, Kanazawa University Graduate School of Medical Sciences, Kanazawa, Japan; 2 Department of Haematology and Oncology, Kanazawa University Hospital, Ishikawa, Japan; Kyushu University, Japan

## Abstract

Natural killer (NK) cell malignancies, particularly aggressive NK cell leukaemias and lymphomas, have poor prognoses. Although recent regimens with L-asparaginase substantially improved outcomes, novel therapeutic approaches are still needed to enhance clinical response. Resveratrol, a naturally occurring polyphenol, has been extensively studied for its anti-inflammatory, cardioprotective and anti-cancer activities. In this study, we investigated the potential anti-tumour activities of resveratrol against the NK cell lines KHYG-1, NKL, NK-92 and NK-YS. Resveratrol induced robust G0/G1 cell cycle arrest, significantly suppressed cell proliferation and induced apoptosis in a dose- and time-dependent manner for all four cell lines. In addition, resveratrol suppressed constitutively active STAT3 in all the cell lines and inhibited JAK2 phosphorylation but had no effect on other upstream mediators of STAT3 activation, such as PTEN, TYK2, and JAK1. Resveratrol also induced downregulation of the anti-apoptotic proteins MCL1 and survivin, two downstream effectors of the STAT3 pathway. Finally, resveratrol induced synergistic effect on the apoptotic and antiproliferative activities of L-asparaginase against KHYG-1, NKL and NK-92 cells. These results suggest that resveratrol may have therapeutic potential against NK cell malignancies. Furthermore, our finding that resveratrol is a bonafide JAK2 inhibitor extends its potential benefits to other diseases with dysregulated JAK2 signaling.

## Introduction

Natural killer (NK) cell malignancies are rare in Western countries but relatively common in Asia. These neoplasms, particularly the aggressive NK cell leukaemia/lymphoma subtype, have poor prognoses [1]–[8]. Even when intensive combination chemotherapies are performed, disease relapse and therapy resistance remain frequent issues. Several L-asparaginase regimens were recently shown to have curative potential but are often associated with serious side effects that can be life-threatening [5], [8]–[11]. Therefore, new therapeutic agents with less toxicity and greater efficacy are of particular interest.

Resveratrol, a natural polyphenol found in red grapes, berries, peanuts and other fruits, has been extensively studied for its antioxidant, anti-aging and anti-inflammatory activities. In addition, *in vivo* and *in vitro* studies showed that resveratrol possesses potent anti-tumour activity against several malignancies. These effects are mediated by targeting molecules involved in the regulation of cell proliferation and survival, such as phosphatase and tensin homologue (PTEN)/Akt, nuclear factor (NF)-κB and signal transducer and activator of transcription 3 (STAT3) [12]–[16]. Constitutive STAT3 activation plays a critical role in the growth and survival of several cancers, including NK neoplasms [17]–[24]. We present the first report of resveratrol efficacy in eliminating NK cell malignancies by inhibiting the Janus kinase 2 (JAK2)/STAT3 pathway, and its notable effectiveness against KHYG-1 cells resistant to L-asparaginase therapy.

## Materials and Methods

### Cell Lines

The NK cell lines NK-92 [20], KHYG-1 [25] (a generous gift from Dr. Y. Isobe at Juntendo University, Tokyo, Japan), NKL [20] (obtained from Dr. M. J. Robertson at the Bone Marrow Transplantation Program, Indiana University, Indianapolis, IN, USA) and NK-YS [20], [26] were cultured in Iscove’s Modified Dulbecco’s Medium supplemented with 20% fetal bovine serum, 100 µg/ml streptomycin, 100 IU/ml penicillin and 100 IU/ml interleukin-2 (Millipore, Temecula, California, USA) at 37°C and 5% CO_2_.

### Reagents

Resveratrol, protease inhibitor cocktail, phosphatase inhibitor cocktail, anti-α tubulin antibody and anti-p53 antibody were purchased from Sigma (Marlborough, MA, USA). L-asparaginase was obtained from ITSI-Biosciences (Johnstown, PA, USA). AG490 was purchased from Merck Millipore (Temecula, California, USA). Antibodies against survivin, myeloid leukaemia cell differentiation protein 1 (MCL1), p21 Waf1/Cip1, p53, cdc2, cdk2, Bcl-2, Bcl-10, cleaved caspase-3 (Asp175), phosphorylated STAT3 (Tyr705), acetylated STAT3 (Lys685), phosphorylated PTEN (Ser380/Thr382/383), phosphorylated tyrosine kinase 2 (TYK2, Tyr1054/1055), phosphorylated Akt (Thr308), phosphorylated JAK1 (Tyr1022/1023), total JAK1, and phosphorylated JAK2 (Tyr1007/1008) were acquired from Cell Signaling Technology (Danvers, MA, USA). Anti-total JAK2 antibody was purchased from GenScript (Piscataway, NJ, USA). Anti-cdk3 antibody was obtained from Genetex (San Antonio, Texas). Anti-STAT3 and anti-murine double minute (Mdm2) antibodies were purchased from Protein Express (Kisarazu, Chiba, Japan) and Acris Antibodies (San Diego, CA, USA), respectively.

### Transient Transfection of STAT3 siRNA

NKL cells were transfected with STAT3 siRNA 100 nM (Cell Signaling Technology) by electroporation using a Bio-Rad Pulser II (Bio Rad, Hercules, CA) as previously described [27]. Nonspecific siRNA (Cell Signaling Technology) was used as a negative control. Protein extraction was performed at 48 h of transfection. Western blotting was used to examine the efficiency of transfection.

### Cell Proliferation Assay

Cells were cultured in the absence or presence of increasing concentrations of resveratrol for 48 h. Cell viability was determined using 3-(4,5-dimethylthiazol-2-yl)-2,5-diphenyltetrazolium bromide (MTT, Roche, Basel, Switzerland) according to the manufacturer’s instructions. Cells were also treated with 50 µM of resveratrol and their proliferation assessed at different exposure times. The inhibitory rate was calculated as follows: inhibitory rate (%) = [(A−B)/A] ×100, where A is the mean optical density of control cells and B is the mean optical density of test sample cells.

### Annexin V Staining

Cells at a density of 5×10^5^/ml were treated with various concentrations of resveratrol, L-asparaginase or AG490 at the indicated times. The percentage of apoptosis was measured by flow cytometry using annexin V-FITC (Invitrogen, Carlsbad, CA, USA) and 7-aminoactinomycin D (BD Pharmingen Biosciences, San Diego, CA, USA) according to the manufacturer’s instructions.

Synergistic effect between resveratrol and L-asparaginase was determined by the cooperative index (CI) based on the Chou-Talalay method [28], [29]. CI = sum of apoptosis of single agent treatment/apoptosis upon combined treatment. When CI<1, CI = 1, and CI>1, the effects were defined as synergistic, additive, and antagonistic respectively.

### Giemsa Staining

Cells were treated with 50 µM of resveratrol for 12, 24 and 48 h, with untreated cells as a control. The cells were then collected, resuspended in phosphate buffered solution and centrifuged using cytospin at 800 rpm for 8 min to adhere on microscopic slides. The cells were then air dried, fixed with methanol, stained with Giemsa (Merck) for 20 min, rinsed in water and air dried again. To evaluate apoptosis, morphological changes were assessed under a light microscope.

### Western Blotting Analysis

Cells were treated with 50 µM of resveratrol for the indicated times. After treatment, whole cell protein samples were prepared by lysing cells in M-PER® Mammalian Protein Extraction Reagent (Pierce, Rockford, IL, USA) supplemented with phosphatase inhibitor cocktail and protease inhibitor cocktail (Sigma) for 30 min at 4°C. 50 µg of total cellular protein was separated by sodium dodecyl sulfate-polyacrylamide gel electrophoresis (SDS-PAGE) and transferred to polyvinylidene fluoride membranes (Millipore). Milk-blocked blots were incubated at 4°C overnight with primary antibodies and then with appropriate horseradish peroxidase-conjugated secondary antibodies (anti-mouse IgG was obtained from KPL, Gaithersburg, MD, USA and anti-rabbit IgG from Vector, Burlingame, CA, USA). Proteins of interest were revealed using West Pico chemiluminescence (Pierce) and viewed in the luminescent image analyser Las-400 Mini (Fujifilm, Tokyo, Japan).

### Flow Cytometry for Cell Cycle Analysis

Cells were treated with 50 µM of resveratrol for 12 h. Cells were then fixed with 70% cold ethanol and analysed after propidium iodide staining. DNA content was determined by FACSCalibur (Becton Dickinson, Franklin Lakes NJ, USA). Data analysis was performed using FlowJo software, version 7.6.3 (Ashland, OR, USA).

### Data Analysis

All data are reported as the mean ± SD of triplicate experiments. Results were analysed using the Student’s *t*-test. All analyses were performed with GraphPad Prism software, version 5.02 (San Diego, CA, USA). A *P-value* <0.05 was considered significant.

## Results

### Effect of Resveratrol on NK Cell Line Proliferation, Viability and Apoptosis

**Figure 1:**
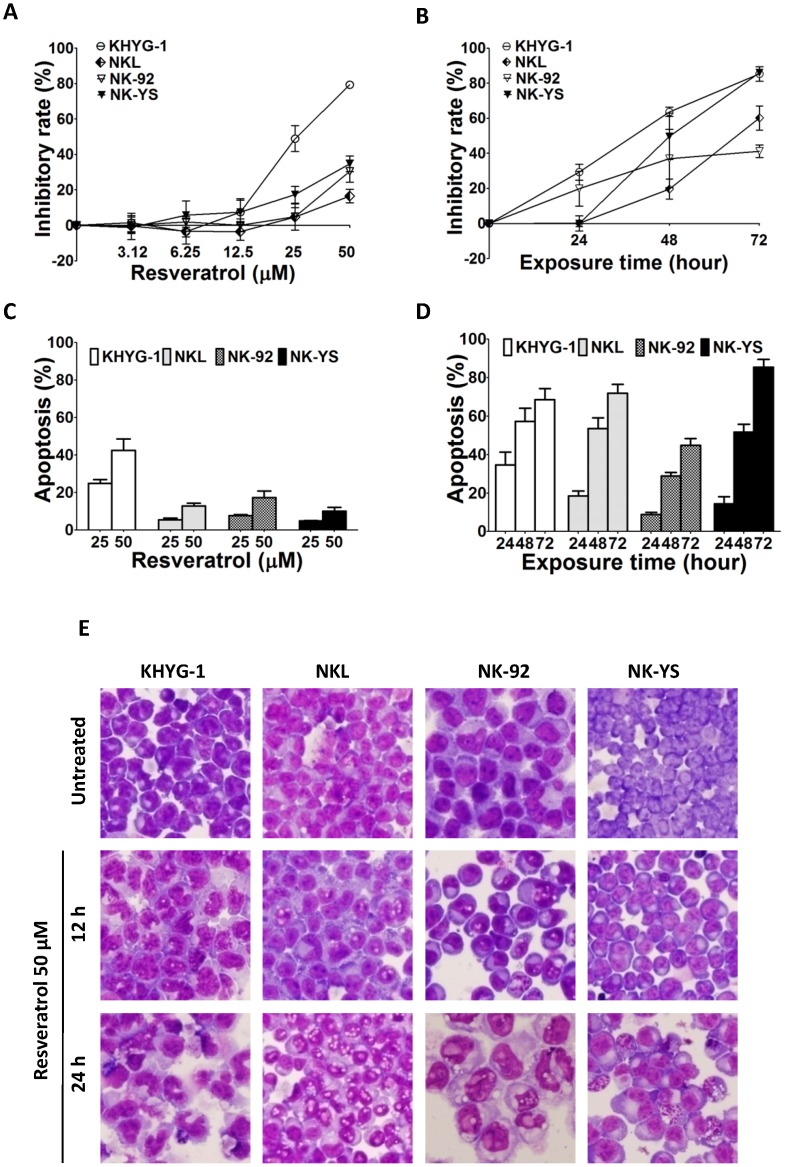
NK cell line growth inhibition and apoptosis induced by resveratrol. Cells were plated in triplicate and treated with increasing concentrations of resveratrol (3.125, 6.25, 12.5, 25 and 50 µM) for 48 h (A) or 50 µM for 24, 48 and 72 h (B). Cell proliferation was then evaluated using the MTT assay. For the apoptosis study, cells (1×10^6^/ml) at 37°C were treated with resveratrol at the indicated concentrations for 24 h (C) or with 50 µM of resveratrol for the indicated times (D). The cells were then incubated with anti-annexin V antibody conjugated to FITC and analysed by flow cytometry. Data represent the drug-induced increase in the percentage of apoptotic cells compared to the respective values observed in parallel control cultures. Results are the mean of data, with error bars representing the SD of triplicate values. (E) Cells were treated with 50 µM of resveratrol for 12 and 24 h and stained with Giemsa to observe morphological changes. The images shown are representative results of three independent experiments.

To investigate its potential anti-tumour activities, malignant KHYG-1, NKL, NK-92 and NK-YS cells were cultured in the presence of resveratrol. In a dose-dependent (Fig. 1A) and time-dependent manner (Fig. 1B), resveratrol significantly inhibited proliferation of all four cell lines. Resveratrol also induced dose-dependent (Fig. 1C) and time-dependent (Fig. 1D) apoptosis in all four lines. KHYG-1 cells were notably the most sensitive to resveratrol (Figs. 1A, 1B, and 1C). To investigate morphological changes induced by resveratrol, cells were stained with Giemsa. As displayed in Figure 1E, cells undergoing resveratrol treatment showed morphological signs of apoptosis such as nuclear condensation [30] but were also preceded by cytoplasmic vacuolisation at 12 hours of treatment.

### Effect of Resveratrol on NK Cell Cycle Phase Distribution

**Figure 2:**
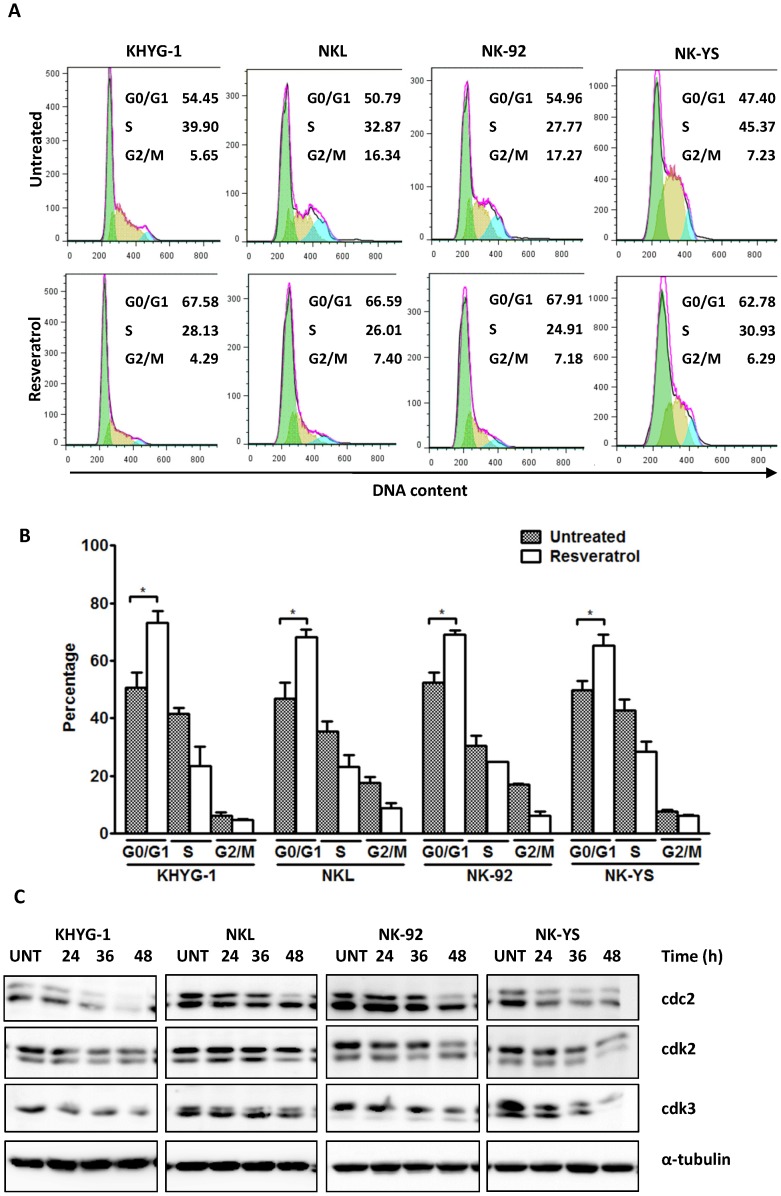
Effect of resveratrol on NK cell cycle distribution. (A) Cells were treated with 50 µM of resveratrol for 12 h and stained with propidium iodide. DNA content was analysed by flow cytometry. The representative results of three independent experiments are shown. (B) Results are the mean of data, with error bars representing the SD of triplicate values. (C) Cells were treated with 50 µM of resveratrol for 24, 36 and 48 h. 50 µg of whole cell extract was prepared for each cell line, separated with SDS-PAGE and subjected to Western blotting with antibodies specific to cdc2, cdk2 and cdk3. The blots were stripped and reprobed with α-tubulin antibody to show equal protein loading. The figures shown are representative results of three independent experiments.*P<0.05.

Because previous reports found that resveratrol modulates the expression of regulatory proteins involved in cell cycle progression [12], [31], [32], its effect on NK cell cycle status was examined. Treatment with 50 µM of resveratrol for 12 hours resulted in a significant accumulation of cells in the G0/G1 phase for all four cell lines (Figs. 2A and 2B) (*P*<0.05). This effect was associated with a resveratrol-induced downregulation of cell-cycle regulator proteins, such as cdc2, cdk2, and cdk3, for all four cell lines (Fig. 2C), a finding consistent with a previous report in Burkkit’s lymphoma cells [33]. This finding suggests that resveratrol anti-tumour activity in malignant NK cells involves G0/G1 cell cycle arrest.

### Effect of Resveratrol on STAT3 and Apoptotic Protein Activity

**Figure 3:**
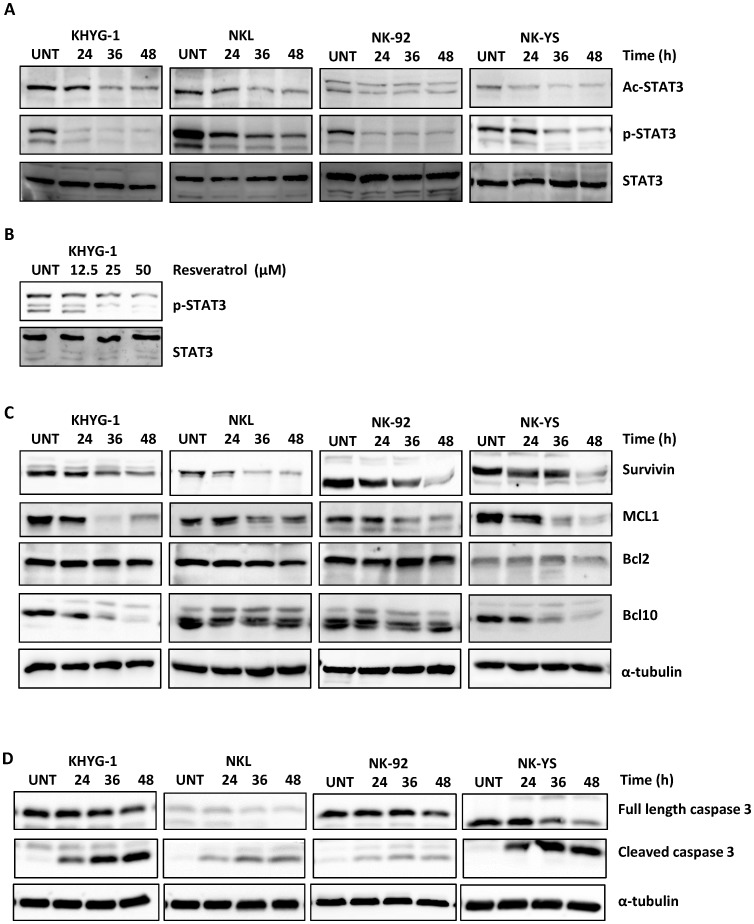
Effect of resveratrol on STAT3 activity and anti-apoptotic proteins in NK cell lines. Cells were treated with 50 µM of resveratrol for 24, 36 and 48 h. 50 µg of whole cell extract was prepared for each cell line, separated with SDS-PAGE and subjected to Western blotting with antibodies specific to acetylated STAT3 (Ac-STAT3), phosphorylated STAT3 (p-STAT3) and STAT3 (A) or MCL1, Bcl-2, Bcl-10 and survivin (C) or cleaved caspase 3 (D). KHYG-1 cells were treated with the indicated concentrations of resveratrol for 24 h (B). The blots were stripped and reprobed with α-tubulin antibody to show equal protein loading. The figures shown are representative results of three independent experiments.

Since STAT3 signaling is required for NK cell survival and proliferation [23] and previous studies showed that resveratrol is a strong STAT3 inhibitor, the effect of resveratrol on STAT3 activation was investigated in malignant NK cells. As displayed in Figure 3A, Western blot studies showed that all NK cell lines constitutively expressed phosphorylated STAT3 at Tyr705. Resveratrol notably had time- and dose-dependent kinetic effects on inhibiting STAT3 phosphorylation in NK cell lines (Figs. 3A and 3B). Acetylated STAT3, which recently attracted attention as a tumour-promoting factor [34], was also constitutively expressed by the four cell lines and had its expression markedly reduced by resveratrol.

The effect of resveratrol on MCL1 and survivin was examined because they mediate anti-apoptotic signals downstream of STAT3 activation, and previous studies showed that malignant NK cells constitutively express these proteins [20], [35], [36]. The effect of resveratrol against other anti-apoptotic proteins including Bcl-2 and Bcl-10 was also investigated. As shown in Figure 3C, resveratrol strongly inhibited MCL1, Bcl-10 and survivin protein expression in all four cell lines. Induction of apoptosis by resveratrol was accompanied by cleavage of caspase 3 in all four cell lines (Fig. 3D). The relations between STAT3 inhibition and cell cycle distribution, apoptosis, MCL1 and survivin were also confirmed in Figure S4 with STAT3 knock down by transient transfection of STAT3 siRNA. These results suggest that STAT3 inhibition plays a role in resveratrol anti-tumour activities against malignant NK cells.

### Downregulation of JAK2 by Resveratrol

**Figure 4:**
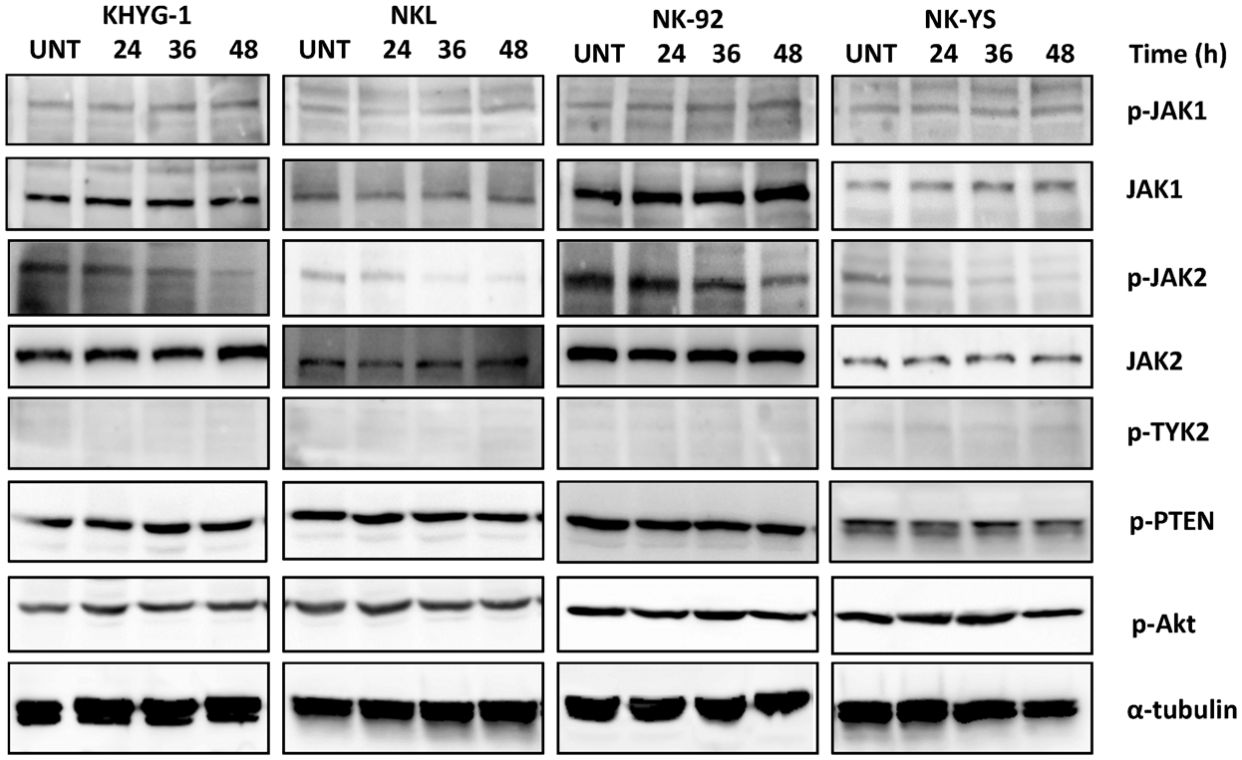
Effect of resveratrol on STAT3 upstream proteins in NK cell lines. Cells were treated with 50 µM of resveratrol for 24, 36 and 48 h. 50 µg of whole cell extract was prepared for each cell line, separated on SDS-PAGE and subjected to Western blotting with antibodies specific to phosphorylated JAK1 (p-JAK1), JAK1, phosphorylated TYK2 (p-TYK2), phosphorylated Akt (p-Akt), phosphorylated PTEN (p-PTEN), phosphorylated JAK2 (p-JAK2) and JAK2. The blots were stripped and reprobed with anti-α-tubulin antibody to show equal protein loading. The figures shown are representative results of three independent experiments.

To elucidate potential mechanisms of resveratrol in STAT3 signaling inhibition, its effect on upstream regulators of STAT3 activation (the JAK family, PTEN and Akt) was examined. Western blot studies showed that while constitutively active JAK1, JAK2, PTEN and Akt were expressed in the four NK cell lines, phosphorylated TYK2 was not detected in any of the cells studied. Resveratrol treatment resulted in consistent inhibition of JAK2 phosphorylation but had no effect on the activation state of JAK1, PTEN, Akt and TYK2 (Fig. 4). Interestingly, chemical inhibition of JAK2 phosphorylation with AG490 vigorously induced apoptosis in all four cell lines (Fig. S1A), and inhibited the expression of STAT3 and its downstream proteins such as MCL1 and survivin (Fig. S1B), thus indicating that inhibition of the JAK2/STAT3 pathway plays a crucial role in the anti-tumour activities of resveratrol against malignant NK cells.

### NK Cell Apoptosis Induced by Resveratrol via a p53-independent Pathway

**Figure 5:**
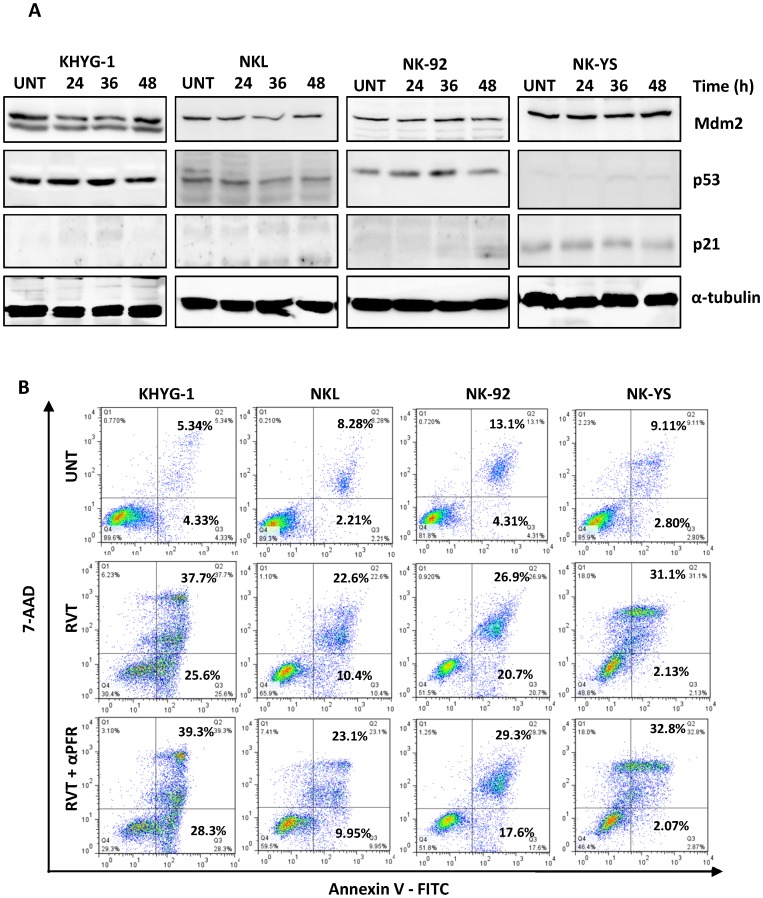
Effect of resveratrol on the p21/p53 axis and Mdm2/p53 axis in NK cell lines. (A) Cells were treated with 50 µM of resveratrol for 24, 36 and 48 h. 50 µg of whole cell extract was prepared for each cell line, separated by SDS-PAGE and subjected to Western blotting with antibodies specific to Mdm2, p53 and p21 antibodies. The blots were stripped and reprobed with anti- α-tubulin antibody to show equal protein loading. (B) Cells (1×10^6^/ml) were incubated with pifithrin α for 2 h in one group before treatment of 50 µM of resveratrol for 24 h. The cells were then incubated with anti-annexin V antibody conjugated to FITC and analysed by flow cytometry to evaluate apoptosis. The figures shown are representative results of three independent experiments.

The role of p53 in resveratrol-induced apoptosis was investigated because previous studies showed that its anti-tumour effects may be mediated in a p53-dependent or p53-independent manner [37]–[39]. Total protein extracts from NK cells treated with resveratrol were prepared, and their expression of total p53, phosphorylated p53, p53 downstream protein p21 and p53 repressor Mdm2 were analysed by Western blot. Resveratrol did not affect protein expression levels in the four cell lines studied (Fig. 5A). In addition, NK cells pre-cultured with or without pifithrin α, a p53 inhibitor which is by itself non-toxic to the NK cell lines (Fig. S2), were equally susceptible to resveratrol-induced apoptosis (Fig. 5B), indicating that functional p53 is not required for resveratrol anti-tumour activity against malignant NK cells.

### Synergistic Effect of Resveratrol Combined with L-asparaginase

**Figure 6:**
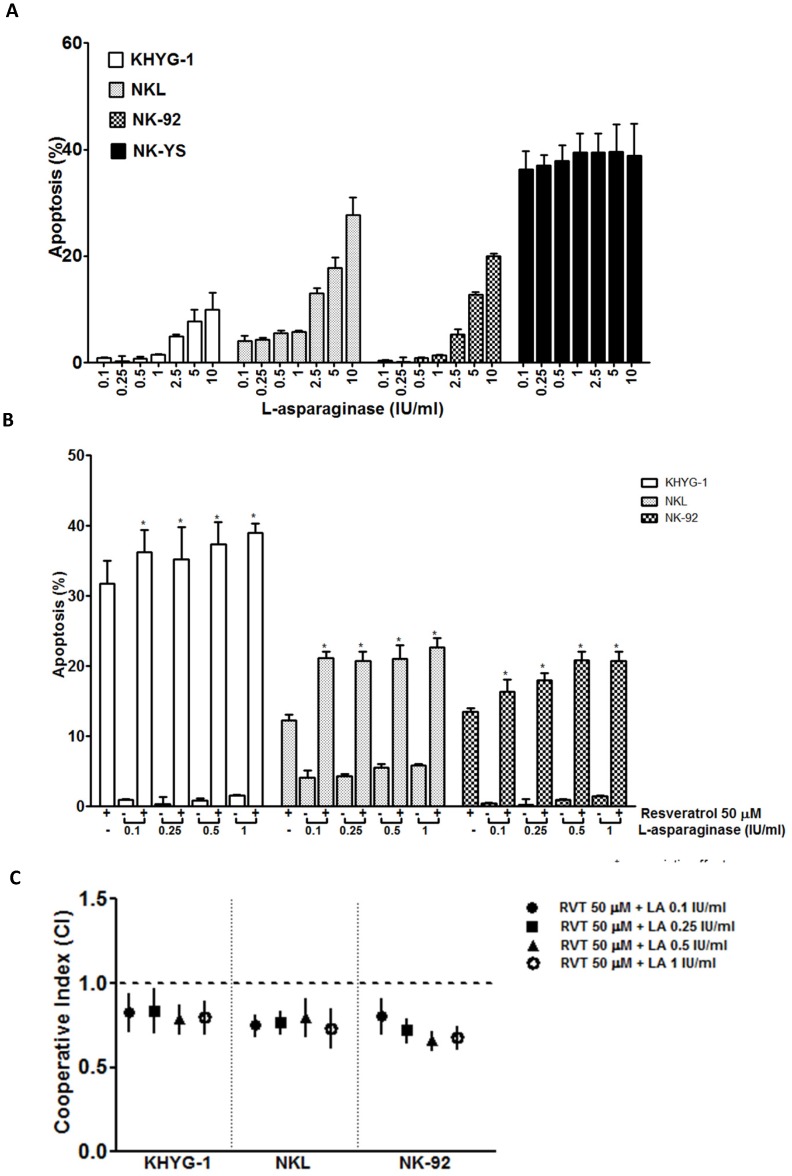
Synergistic effect of resveratrol combined with L-asparaginase in NK cell lines. Cells (1×10^6^/ml) were treated with L-asparaginase (A) or resveratrol plus L-asparaginase (B) at the indicated concentrations for 24 h. The cells were then incubated with anti-annexin V antibody conjugated to FITC and analysed by flow cytometry to evaluate apoptosis. The CI was calculated for each condition (C). The results are the mean of data, with error bars representing the SD of triplicate values. Data represent the drug-induced increase in the percentage of apoptosis cells compared to the respective values observed in parallel control cultures. When CI<1, CI = 1, and CI>1, the effects were defined as synergistic, additive, and antagonistic respectively.

*In vitro* and clinical studies found that L-asparaginase is a promising anticancer agent against NK malignancies [39], [40]. To examine potential synergistic activity between resveratrol and L-asparaginase against NK cell neoplasms, the four lines were treated with L-asparaginase alone or with resveratrol and assessed by flow cytometry to measure cell apoptosis. Consistent with previous reports [2], [40], L-asparaginase sensitivity varied among the cell lines studied; NK-YS was highly sensitive, NK-92 and NKL were moderately sensitive and KHYG-1 cells were resistant to L-asparaginase (Fig. 6A). In contrast to resveratrol, apoptosis induced by L-asparaginase was not preceded by autophagy-like changes (Fig. S3), indicating that the anti-tumour activities of those compounds are mediated by different mechanisms. Remarkably, L-asparaginase with resveratrol resulted in a synergistic cytotoxic effect against KHYG-1, NKL and NK-92 cells (Figs. 6B and 6C). The synergistic effect was not observed in NK-YS cells (data not shown) presumably because of the extremely high sensitivity of this cell line to L-asparaginase (Fig. 6A).

## Discussion

This study demonstrated that resveratrol, a naturally occurring polyphenol, strongly inhibited the growth and survival of malignant NK cells. Mechanism studies revealed that resveratrol inhibits constitutively active STAT3 signaling, leading to downregulation of the anti-apoptotic proteins MCL1, Bcl-10, and survivin. These events contributed to cell cycle arrest at G0/G1 and eventual loss of viability due to apoptosis.

STAT proteins function as downstream effectors of growth factor receptors and cytokine signals that participate in vital processes such as cell proliferation and apoptosis [41]. STAT3 is constitutively activated in human solid tumour cancers such as breast cancer and prostate cancer, as well as myelomas and NK cell lymphomas [21], [23], [42]–[46]. Aberrant STAT3 signaling in malignant cells thus represents a promising therapeutic target. Our findings show that resveratrol effectively inhibits STAT3 in malignant NK cells, which agrees with other studies that found the polyphenol was an efficacious inhibitor of constitutively active STAT3 in malignant cells [47], [48]. Our findings that resveratrol inhibited STAT-3 as well as its downstream anti-apoptotic proteins MCL-1 and survivin are also consistent with its effect on EBV-infected B cells in our previous study [49]. The expression of the anti-apoptotic protein Bcl-2 was not affected by the resveratrol-induced JAK2 inhibition which is consistent with the report by Tsutsui et al [20], showing that JAK2 inhibition with AG490 does not down-regulate Bcl-2 protein in malignant NK cells.

A previous study showed that leukaemic cells lacking functional p53 are L-asparaginase resistant [50]. This is consistent with our finding that KHYG-1 cells with a *P53* mutation [25] were resistant to L-asparaginase. Remarkably, the other three cell lines NKL, NK-92 and NK-YS, all of which express wild type *P53* protein as reported in previous studies [51], [52], were more sensitive to L-asparaginase treatment. Interestingly, KHYG-1 cells were the most sensitive to resveratrol treatment of the four NK cell lines. Efficient induction of apoptosis by resveratrol in NK cells lacking functional p53 is consistent with a report that resveratrol induced both p53-dependent and p53-independent apoptosis [53].

In contrast to previous studies where resveratrol enhanced the function of tumour suppressor PTEN and inhibited activated Akt in tumour cells [47], [54], resveratrol treatment in this study did not affect expression of JAK1, PTEN, TYK2 and Akt. The inhibitory effect of resveratrol on STAT3 activation was mediated by inhibition of phosphorylated JAK2 in all four NK cell lines. This is consistent with our findings that the cell lines underwent apoptosis and STAT3 signaling was strongly inhibited when exposed to the JAK2 inhibitor AG490 (Fig. S1), as well as a previous report on NK-YS cells treated with AG490 [20]. Resveratrol-induced JAK2 inhibition is further supported by our findings showing reduction of phosphorylated JAK2 and resveratrol induced apoptosis in HEL cells (data not shown), a myeloid cell line with a *JAK2* activation mutation [55]. These findings collectively suggest that resveratrol is a JAK2 inhibitor with considerable clinical relevance, given that dysregulated JAK2 activity is involved in the pathogenesis of hematological diseases such as myeloproliferative neoplasms [56].

One intriguing finding of this study was that resveratrol-induced apoptosis was preceded by cellular morphological changes characterized by accumulation of autophagic vacuoles in the cytoplasm. The absence of these changes in NK cells treated with L-asparaginase (Fig. S3) supports the notion that these compounds exert their anti-tumour effects through different mechanisms. Apoptotic cell death and autophagic cell death have been broadly accepted as two separate mechanisms of programmed cell death, although recent reports suggest that autophagic vacuole accumulation could precede apoptotic cell death [30]. Interestingly, similar morphological changes have been reported in human colorectal DLD1 cancer cells treated with resveratrol [57].

Previous studies revealed that resveratrol is a potent activator of sirtuin 1 (SIRT1) [58]–[62] and that SIRT1 mediates downregulation of acetylated STAT3 in various cellular systems [63], [64]. This is consistent with our finding that acetylated STAT3 decreased in NK cell lines treated with resveratrol and supports the concept that STAT3 deacetylation is a contributing anti-tumour property. Although this study did not investigate downstream effects of resveratrol-induced STAT3 deacetylation, a recent report demonstrated that acetylated STAT3 methylates the promoter region of tumor suppressor genes and results in gene silencing that promotes tumour cell proliferation. The same study also showed that inhibition of STAT3 acetylation by resveratrol results in promoter demethylation, restoring the expression of tumour suppressor genes [34].

In conclusion, our data indicate that resveratrol has potent anti-tumour effects against malignant NK cells that are mediated through inhibition of constitutively active STAT3 signals due to blocking JAK2 kinase. This study is the first to demonstrate that resveratrol is a potent JAK2 inhibitor. As a result, resveratrol may have therapeutic potential against not only NK neoplasms but also haematological disorders where dysregulated JAK2 signaling plays a critical role in pathogenesis.

## Supporting Information

Figure S1 **Effect of JAK2 inhibitor AG490 in NK cell lines.** Cells (1×10^6^/ml) were treated with 100 µM of AG490 for 24 and 48 h. The cells were then incubated with anti-annexin V antibody conjugated to FITC and analysed by flow cytometry to evaluate apoptosis. (B) Cells were treated with 100 µM of AG490 for 24, 36 and 48 h. 50 µg of whole cell extract was prepared for each cell line, separated by SDS-PAGE, and subjected to Western blotting with antibodies specific to phosphorylted STAT3 (p-STAT3), STAT3, MCL1, and survivin. The blots were stripped and reprobed with anti-α-tubulin antibody to show equal protein loading. Figures shown are representative results of three independent experiments.
(TIF)

Figure S2 **Apoptotic effect of a p53 inhibitor pifithrin α in NK cell lines.** Cells (1×10^6^/ml) were treated with 30 µM of pifithrin α for 24 h. The cells were then incubated with anti-annexin V antibody conjugated to FITC and analyzed by flow cytometry to evaluate apoptosis. Figures shown are representative results of three independent experiments.
(TIF)

Figure S3 **Effect of L-aparaginase on NK cell morphology.** Cells were treated with 10 IU/ml of L-asparaginase for 12 and 24 h and stained with Giemsa to observe morphological changes. The images shown are representative results of three independent experiments.
(TIF)

Figure S4 **Effect of STAT3 siRNA on cell cycle progression, apoptosis and STAT3 signaling in NK cell lines.** Cell cycle analysis (A), Annexin V staining (B) and Western blotting with antibodies specific to phosphorylated STAT3, STAT3, MCL1, and survivin (C) were performed at 48 h after transfection with STAT3 siRNA. The representative results of two or more independent experiments are shown.
(TIF)
