# Supplementary figures and images for: Resveratrol Induces Cell Cycle Arrest and Apoptosis in Malignant NK Cells via JAK2/STAT3 Pathway Inhibition
Source: PLoS One. 2013 Jan 25;8(1):e55183. doi: 10.1371/journal.pone.0055183 (PMC3555980; doi:10.1371/journal.pone.0055183)

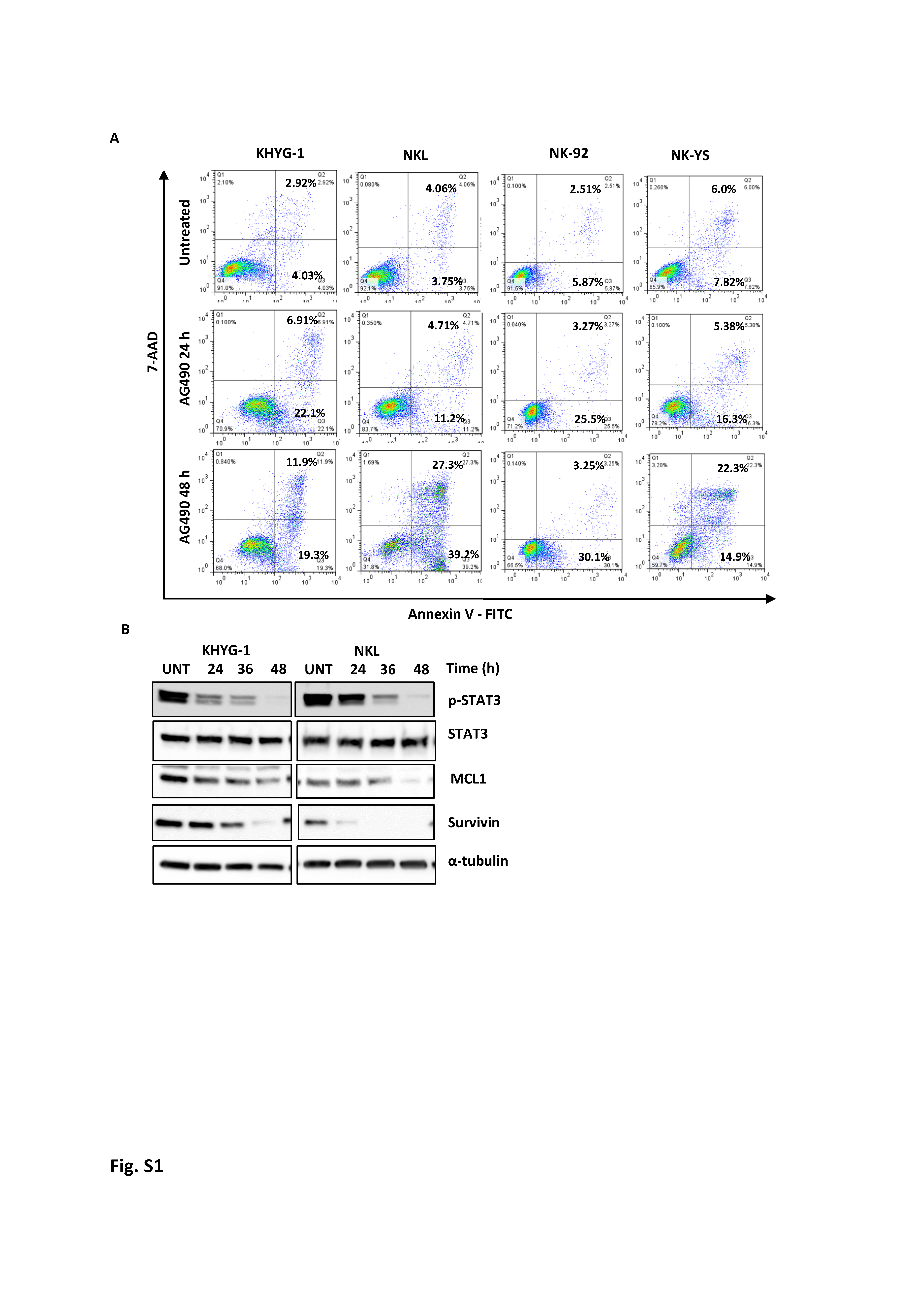

Supplement: Figure S1 — Effect of JAK2 inhibitor AG490 in NK cell lines. Cells (1×10^6^/ml) were treated with 100 µM of AG490 for 24 and 48 h. The cells were then incubated with anti-annexin V antibody conjugated to FITC and analysed by flow cytometry to evaluate apoptosis. (B) Cells were treated with 100 µM of AG490 for 24, 36 and 48 h. 50 µg of whole cell extract was prepared for each cell line, separated by SDS-PAGE, and subjected to Western blotting with antibodies specific to phosphorylted STAT3 (p-STAT3), STAT3, MCL1, and survivin. The blots were stripped and reprobed with anti-α-tubulin antibody to show equal protein loading. Figures shown are representative results of three independent experiments. (TIF) [file pone.0055183.s001.tif]

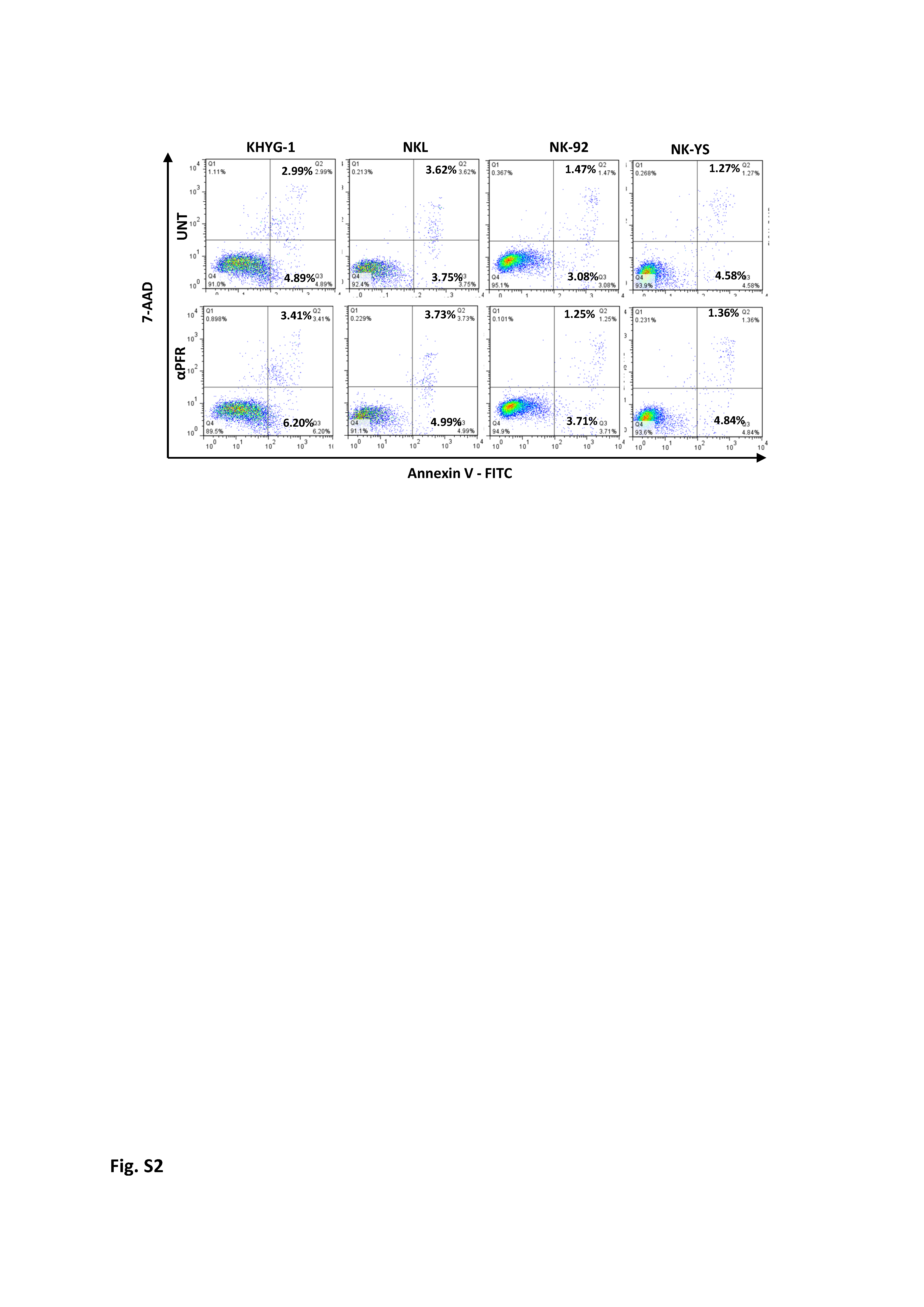

Supplement: Figure S2 — Apoptotic effect of a p53 inhibitor pifithrin α in NK cell lines. Cells (1×10^6^/ml) were treated with 30 µM of pifithrin α for 24 h. The cells were then incubated with anti-annexin V antibody conjugated to FITC and analyzed by flow cytometry to evaluate apoptosis. Figures shown are representative results of three independent experiments. (TIF) [file pone.0055183.s002.tif]

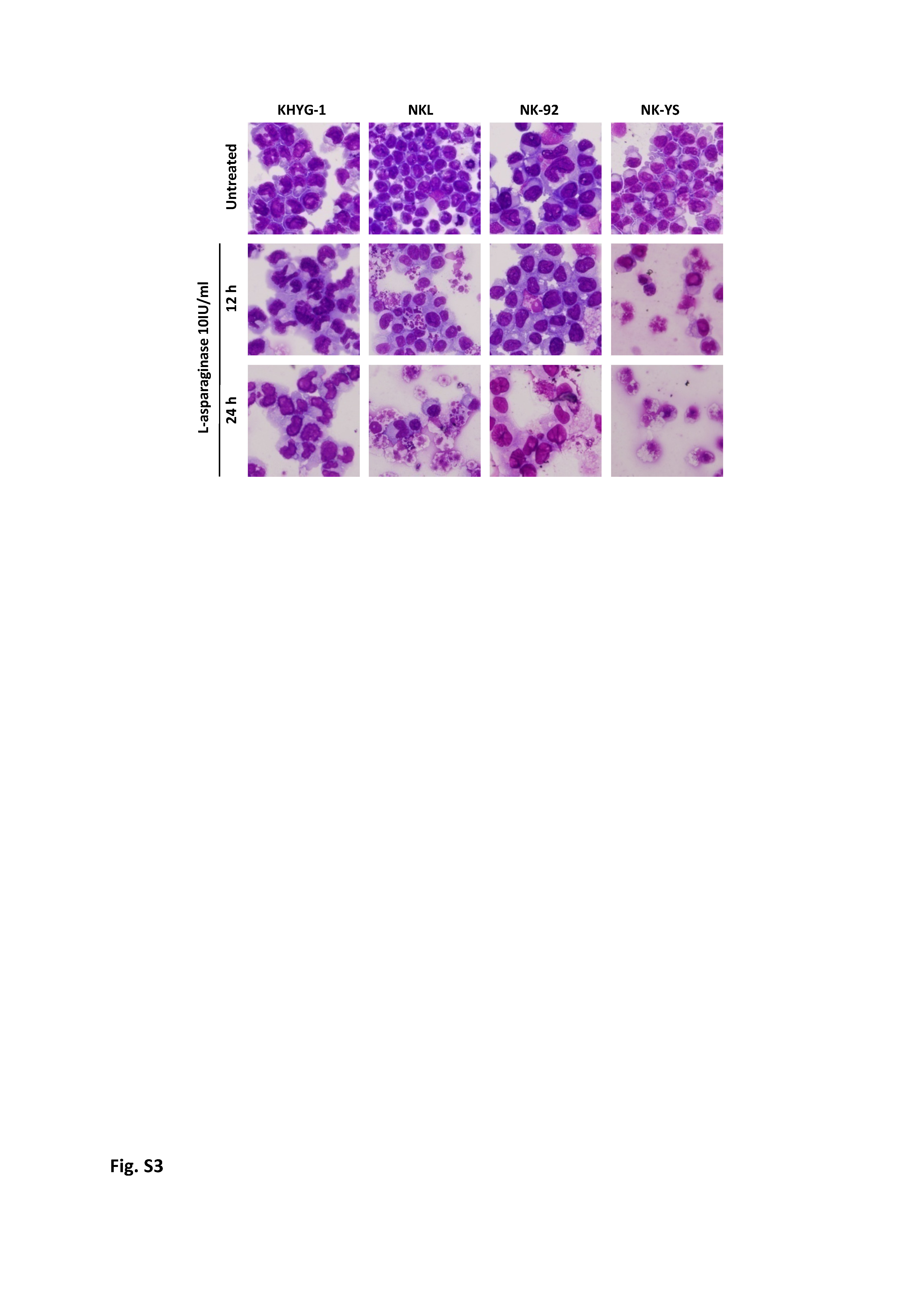

Supplement: Figure S3 — Effect of L-aparaginase on NK cell morphology. Cells were treated with 10 IU/ml of L-asparaginase for 12 and 24 h and stained with Giemsa to observe morphological changes. The images shown are representative results of three independent experiments. (TIF) [file pone.0055183.s003.tif]

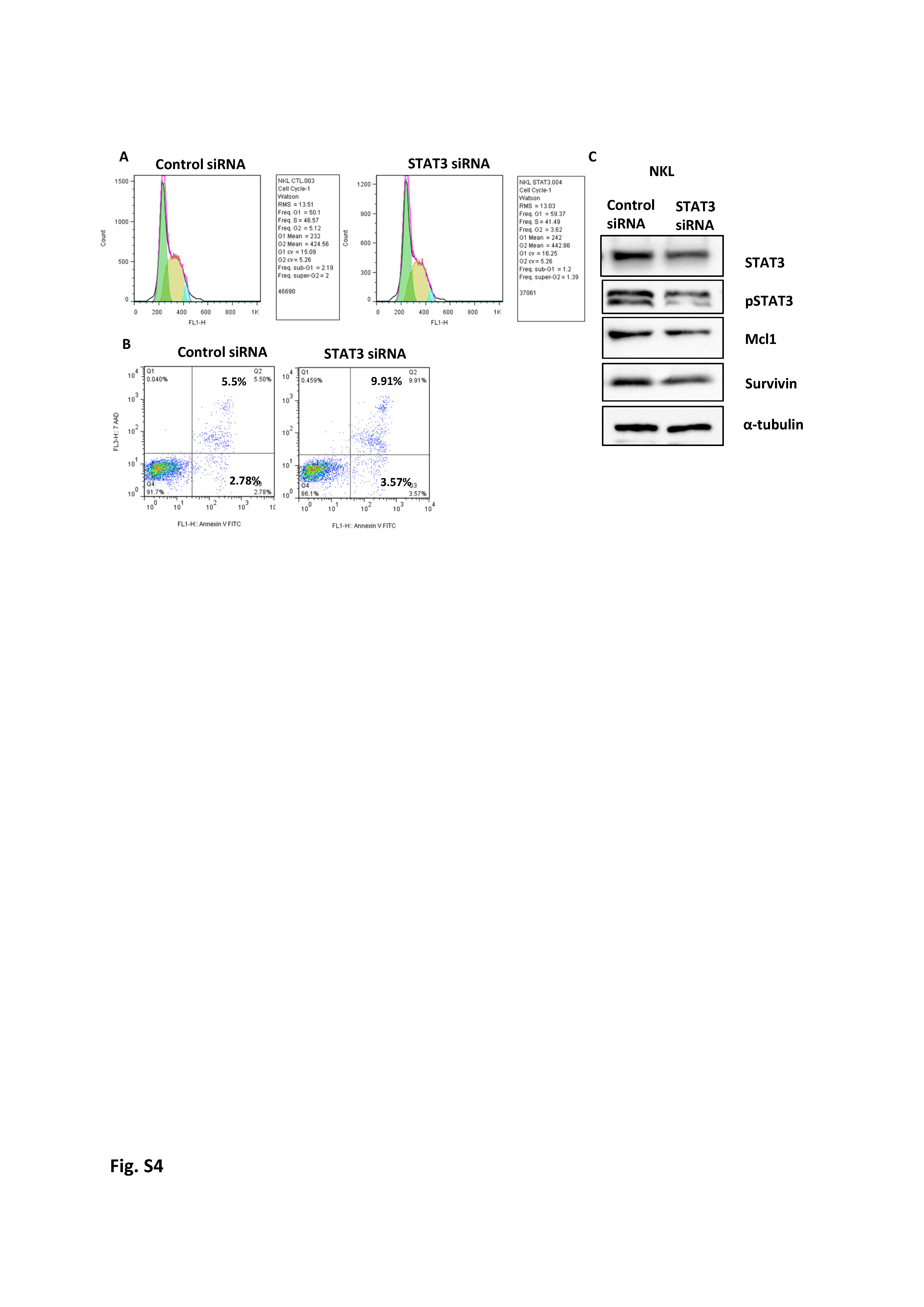

Supplement: Figure S4 — Effect of STAT3 siRNA on cell cycle progression, apoptosis and STAT3 signaling in NK cell lines. Cell cycle analysis (A), Annexin V staining (B) and Western blotting with antibodies specific to phosphorylated STAT3, STAT3, MCL1, and survivin (C) were performed at 48 h after transfection with STAT3 siRNA. The representative results of two or more independent experiments are shown. (TIF) [file pone.0055183.s004.tif]
